# Total versus hemithyroidectomy for small unilateral papillary thyroid carcinoma

**DOI:** 10.3892/ol.2013.1765

**Published:** 2013-12-18

**Authors:** DANIA HIRSCH, SIGAL LEVY, GLORIA TSVETOV, ILAN SHIMON, CARLOS BENBASSAT

**Affiliations:** 1 Institute of Endocrinology, Rabin Medical Center, Beilinson Hospital, Petach Tikva 49100, Israel; 2 Sackler Faculty of Medicine, Tel Aviv University, Tel Aviv 6997801, Israel; 3 Academic College of Tel Aviv-Yaffo, Tel Aviv 6818211, Israel; 4 Sackler Faculty of Exact Sciences, Tel Aviv University, Tel Aviv 6997801, Israel

**Keywords:** thyroid surgery, thyroid cancer, thyroidectomy, outcome, thyroid

## Abstract

The correct approach to treat low-risk intrathyroidal papillary thyroid carcinoma (PTC) is controversial. Specific authors advocate unilateral thyroidectomy to minimize perioperative morbidity. The purpose of the present study was to determine an effective treatment strategy for patients with small unilateral papillary thyroid. This was a retrospective comparative analysis of 161 patients with PTC treated between 2001–2010; 60 consecutive patients following hemithyroidectomy and 101 patients following total thyroidectomy. Only patients with preoperatively-predicted localized unilateral disease were included. No between-group difference was identified in the rate of permanent surgical complications. In total, 36 hemithyroidectomy patients (60%) exhibited benign thyroid nodules in the contralateral lobe on preoperative ultrasound; this factor was found to positively correlate with the performance of ≥1 fine needle aspirations (FNAs) during follow-up. In addition, 47 hemithyroidectomy patients (78.3%) were prescribed thyroxine postoperatively. The hemithyroidectomy patients visited the endocrine clinic significantly less frequently than the total thyroidectomy patients (P=0.01), but were referred more often for neck ultrasound (P=0.03) and FNA (P<0.001). In addition, an increased number of patients in the hemithyroidectomy group were reoperated for suspected recurrent/persistent disease (P=0.06). Results of this retrospective study indicate that hemithyroidectomy for small unilateral PTC is associated with a significant follow-up burden and provides no clear patient benefit.

## Introduction

There has been a long unresolved debate in the literature with regard to the optimal initial treatment for unilateral papillary thyroid cancer (PTC), including the extent of thyroidectomy (1–4). In cases identified preoperatively, the rationale for total thyroidectomy includes the frequent multifocal nature of the disease, the ability to reduce the risk of recurrence and to allow radioactive iodine treatment and the greater ease of monitoring by whole-body radioiodine scans and serum thyroglobulin levels. This approach has been supported in two previous studies by Mazzaferri *et al* (5,6), which indicated that total thyroidectomy reduced the risk of PTC recurrence and apparently improved survival rates. A more recent large study analyzing data from the National Cancer Data Base between 1985 and 1998 confirmed these observations showing that in patients with tumors measuring >1 cm, total thyroidectomy was associated with a lower recurrence and improved survival rates compared with lobectomy (7). The American Thyroid Association (8) and the European Thyroid Cancer Task Force (9) recommend total thyroidectomy for the majority of PTC cases. However, for small (≤1 cm) solitary, well-differentiated intrathyroidal tumors, less than total or near-total thyroidectomy may be sufficient, particularly when there is no definite diagnosis of PTC prior to surgery.

Although PTC is associated with a low intrinsic mortality, it requires life-long follow-up care. There are clear guidelines with regard to the life-time surveillance of low risk patients following total thyroidectomy (8,9). However, for patients following lobectomy or hemithyroidectomy, the regimen is vague, mainly since there are no means of definitively excluding the presence of PTC in the remaining lobe. Although periodic serum thyroglobulin measurements are recommended in these patients, together with neck ultrasound, the necessary frequency of these assessments has not been defined (8,9).

The aim of the present study was to evaluate and compare the follow-up regimen and outcome of patients treated by hemithyroidectomy or total thyroidectomy for intrathyroidal unilateral PTC at a single medical center.

## Patients and methods

### Patient characteristics

The study was approved by the institutional review board of Rabin Medical Center (Petach Tikva, Israel). The study group consisted of consecutive patients following hemithyroidectomy for PTC who were treated and followed at the Endocrine Institute of Rabin Medical Center (Petach Tikva, Israel), a tertiary university-affiliated hospital, between 2001 and 2010. The control group consisted of consecutive patients with predicted unilateral localized PTC at presentation who were treated with total thyroidectomy (in one or two stages) and followed at the same institute during the same time. Patients with known regional or distant metastases were excluded. The individual medical records were reviewed for the following data: Age, gender, preoperative neck ultrasound observations, pathological observations for tumor size and extension, surgical complications, postoperative levels of thyroid-stimulating hormone (TSH), thyroglobulin and thyroglobulin antibodies and treatment with thyroxine pre- and postoperatively (yes/no). In addition, the number of postoperative patient visits to the endocrine clinic, postoperative blood tests for thyroid function and/or thyroglobulin levels, postoperative neck ultrasounds and fine needle aspirations (FNAs), were documented. For the control group, data on TNM staging of the thyroid cancer according to the American Joint Committee on Cancer (sixth edition), central compartment lymph node dissection (yes/no) and radioactive iodine treatment (yes/no), were also collected. Thyroid function tests for patients who became pregnant during follow-up were excluded. In addition, all patients who underwent a second neck surgery >1 year after their initial surgery, due to suspected PTC persistence/recurrence in the thyroid remnant tissue or cervical lymph nodes, were identified. Patients who required completion thyroidectomy <1 year following hemithyroidectomy due to adverse pathological observations in the first surgery were excluded from the hemithyroidectomy group and included in the total thyroidectomy group.

### Clinical comparisons

The clinical data and follow-up regimens were compared between the study and control groups. In the hemithyroidectomy group, the correlation between the demographic and clinical data and the number of patient follow-up visits, thyroid laboratory tests, neck ultrasound scans and FNAs, was determined.

### Statistical analysis

The independent t-test was used to evaluate between-group differences and Pearson correlation or the χ^2^ test were used to evaluate the relationship between clinical characteristics and follow-up surveillance in the hemithyroidectomy group.

## Results

### Patient analysis

The final analysis included 161 patients with preoperatively predicted localized unilateral PTC; 60 patients were treated with hemithyroidectomy and 101 patients with total thyroidectomy. The patient clinical characteristics are shown in Table I. No significant between-group differences were identified in age or gender distribution or duration of follow-up. The median duration of follow-up was 4.4 years in the hemithyroidectomy group and 4.6 years in the total thyroidectomy group.

In the hemithyroidectomy group, 36 patients (60%) exhibited nodules (2–28-mm in diameter) in the contralateral (non-operated) lobe on the preoperative neck ultrasound scan. Results on preoperative FNA studies were available in 52 hemithyroidectomy patients. The observations were diagnostic for PTC in 38 patients, suspicious for follicular neoplasm in 9 patients and benign in 5 patients; in an additional 2 patients FNA was not performed prior to surgery. In the total thyroidectomy group, preoperative FNA results were available for 97 patients. The observations were diagnostic for PTC in 56 patients, suspicious for follicular neoplasms in 34 patients and benign in 7 patients; in an additional 2 patients, FNA was not performed and the observation of PTC was incidental. Contralateral preoperatively aspirated nodules were negative for malignancy in the two groups.

### Surgical complications

Permanent complications of surgery included recurrent laryngeal palsy in 2 patients in each group and hypoparathyroidism in 3 patients in the total thyroidectomy group and 1 patient in the hemithyroidectomy group. In the latter patient, the hypoparathyroidism developed following a second surgery performed 2 years after the first, due to suspicious cytological observations in the remnant thyroid lobe. The difference in permanent complications among the groups was not statistically significant.

In the hemithyroidectomy group, thyroxine treatment was administered to 9 patients (15%) preoperatively and 47 patients (78.3%) postoperatively, including 38 patients who were initially administered with thyroxine postoperatively. Among these 38 patients, 15 acquired hypothyroidism following surgery and 19 received LT4 prophylactically with no evidence of hypothyroidism. In the remaining 4 patients, no information on postoperative thyroid function tests were available.

### Pathological examination

On pathological examination, 54 patients in the hemithyroidectomy group (90%) exhibited one focus of PTC measuring <10 mm, 3 patients (5%) exhibited tumors measuring 13–19 mm and 3 patients (5%) exhibited minimal extrathyroid extension. In the total thyroidectomy group, 43 patients (42.5%) exhibited multifocal disease. Among these patients, 35 exhibited bilateral foci of PTC and 8 exhibited ≥2 foci of PTC in one lobe of the thyroid with no foci of PTC in the contralateral lobe. In addition, despite the preoperative absence of neck lymphadenopathy on ultrasound, 14 patients in the total thyroidectomy group (13.8%) were found to exhibit metastatic cervical lymph nodes at surgery, which were removed.

### Analysis of disease stage

In the total thyroidectomy group, 70 patients (69.3%) exhibited stage 1 disease, 9 patients (8.9%) exhibited stage 2, 20 patients (19.8%) exhibited stage 3 and 2 patients (1.9%) exhibited stage 4a. In total, 99 patients in this group (98%) received adjuvant radioactive iodine treatment at a dose of 30–150 mCi. In addition, 5 patients in the total thyroidectomy group exhibited evidence of recurrent/persistent disease: 1 patient was underwent a second surgery for metastatic lymph nodes 4 years following the initial surgery and received an additional 2 treatments with radioactive iodine; 2 received a second radioactive iodine treatment with 150 mCi; and 2 did not receive further surgical or radioactive iodine treatment.

Overall, 4 patients (6.6%), initially treated with hemithyroidectomy, underwent completion thyroidectomy >1 year later due to cytological observations compatible with PTC in the remnant lobe. A second neck surgery was more frequent in the hemithyroidectomy than the total thyroidectomy group (6.6 vs. 1%; P=0.06).

### Patient follow-up

Following total thyroidectomy, the patients visited the endocrine clinic significantly more often than the hemithyroidectomy patients (P=0.016). These included visits scheduled for planning radioactive iodine treatment, which were relevant almost exclusively to the total thyroidectomy patients. However, patients following hemithyroidectomy were referred significantly more often for neck ultrasound (P=0.03) and FNAs (P=0.001). Specifically, 16 patients in the hemithyroidectomy group (26.6%) underwent 1 FNA and 15 patients (25%) underwent 2–5 FNAs, whereas, in the total thyroidectomy group, 10 patients (9.9%) underwent one FNA and only 1 patient (0.99%) underwent 2 FNAs. No significant difference was identified in the frequency of thyroid blood tests between the two groups.

Within the hemithyroidectomy group, the parameters that were found to positively correlate with the performance of repeated FNAs were the presence and size of nodules in the remnant lobe. No correlation was identified between plasma TSH, thyroglobulin levels or receipt of thyroxine treatment and the frequency of laboratory thyroid tests, ultrasound scans or FNAs during follow-up, or with the requirement for reoperation.

## Discussion

In the present study, a significant percentage of the patients with low-risk PTC, who were initially treated with hemithyroidectomy, were found to require a more intensive follow-up regimen than patients with larger tumors or more advanced disease that were treated with total thyroidectomy and radioactive iodine. Although only a small minority of the hemithyroidectomy group exhibited evidence of recurrent/persistent disease, a higher percentage compared with the total thyroidectomy group required a repeat surgery due to suspected persistent/recurrent disease.

It is reasonable to assume that these factors negatively affect the quality of life of the patient. Previous studies have shown that health-related quality of life, in terms of anxiety, depression and fatigue, are similar in the first 12-postoperative months in patients with PTC treated with total or less-than-total thyroidectomy (10). However, this decreases significantly in the long-term, even in disease-free survivors of differentiated thyroid cancer (11,12). Using a mathematical model of decision analysis, Esnaola *et al* (13) previously found that total thyroidectomy maximized quality-adjusted life expectancy compared with thyroid lobectomy in low- and high-risk patients with PTC.

In addition, the high number of sonographic and cytological tests have economic implications, with a higher annual follow-up cost for patients following hemithyroidectomy than total thyroidectomy. Previously, Shrime *et al* (14) compared the 20-year cost-effectiveness of hemithyroidectomy with total thyroidectomy in the management of small PTCs in low-risk patients, using cost estimates for initial surgery, follow-up and treatment. The authors found that despite the greater initial cost of total thyroidectomy, in the long term, hemithyroidectomy was associated with an added cost between $300 and $3,000, mainly due to the requirement of secondary operative procedures.

Whilst the majority of the international literature endorses total thyroidectomy for unifocal small PTCs, several previous studies have described papillary thyroid microcarcinoma (PTMC) as an almost benign disease that may be treated using a less aggressive paradigm. In 2003, Ito *et al* found that in 162 patients with diagnosed PTMC who had received observational follow-up alone for ≤6 years, ~70% of cases showed no change in tumor size or regression. The authors summarized that it may be possible to conduct follow-up in these patients with serial imaging and examinations alone if the patient consents to such treatment (15). In a study published in 2008, Siassakos *et al* performed follow-up for 6 years in patients who exhibited PTMC that had been identified incidentally on histopathological studies of thyroid lobectomy specimens. No recurrence, metastases or mortalities were noted. The authors concluded that in this sample of patients with PTMC, no further surgical or radiotherapeutic intervention was warranted (16).

Therefore, the hemithyroidectomy group of the current study is most likely representative of a number of similar patients in various medical centers. Although 60% of the current group of patients exhibited visible thyroid nodules in the contralateral lobe prior to surgery, this observation is also not likely to be exceptional. The reported rate of asymptomatic thyroid nodules was 50% in a previous autopsy study (17) and 67% in a prospective study performed in North America (18). In addition, with predicted improvements in ultrasound resolution over time, the incidence of detected thyroid nodules is likely to increase. Due to the retrospective nature of the present study, the indication for hemithyroidectomy in cases of bilateral nodular goiter is usually difficult to assess and may only be hypothesized. Possible indications may include benign cytology of the nodules in the non-operated lobe, the appearance of permanent recurrent nerve palsy following hemithyroidectomy or the absence of a definite diagnosis of PTC prior to surgery. In fact, these are extremely common scenarios and the decision on the extent of surgery in such cases is always a difficult one, particularly when small nodules with a benign cytology are present in the contra lateral lobe.

In the current study, the analysis of the demographic and clinical factors that may predict the requirement for repeated tests following the initial surgery, revealed that the presence and size of the nodules in the contralateral thyroid lobe prior to hemithyroidectomy were found to correlate with the performance of a high number of FNAs during follow-up.

A potential argument in favor of hemithyroidectomy for small PTCs is the lower incidence of surgical complications. However, to date, there have been no randomized, prospective trials supporting this concept. In a previous statistical study, Udelsman *et al* (19) concluded that to compare the permanent complication rates of these two procedures in a controlled prospective study, researchers are likely to require 12,000 patients with PTC. The authors calculations were based on the assumption that a significant percentage of patients following initial thyroidectomy require a second total thyroidectomy, which has a reportedly higher complication rate than initial total thyroidectomy. In the current study, no significant difference was identified in the rate of permanent complications between the hemithyroidectomy and total thyroidectomy groups. Similar results have been previously reported by other authors (20).

An additional common argument favoring hemithyroidectomy for PTC is that performing this partial surgery may possibly spare the requirement for life-long thyroxine treatment. Nevertheless, in the present study, the majority of the patients in the hemithyroidectomy group were administered thyroxine following surgery.

It has recently been reported that, at present, the most commonly occurring thyroid cancer in the United States, in patients >45 years old, is PTMC (21). Accordingly, it has been predicted that hemithyroidectomy is likely to be performed more frequently. However, the results of the current study suggest that a more appropriate selection of patients for hemithyroidectomy may reduce the uncertainty concerning disease persistence and consequently, the requirement for continuous ultrasonographic and particularly cytological surveillance of the remnant lobe. It may also, as indicated in the literature, reduce the recurrence rate, the requirement for a second surgery and patient anxiety concerning the examinations, as well as the possibility of recurrence.

In summary, patients with a small PTC who undergo hemithyroidectomy alone are prone to a significant follow-up burden and risk of reoperation, with no clear benefit of the limited surgical procedure. This is particularly relevant in patients with bilateral thyroid nodules. In the era of personalized medicine, these observations must be taken into consideration by surgeons planning initial thyroid surgery, providing an additional argument in favor of total or near total thyroidectomy in patients with PTMC or predicted benign multinodular goiter.

## Figures and Tables

**Table I:** Clinical characteristics and follow-up procedures of patients with differentiated thyroid cancer treated by total versus hemithyroidectomy.

Parameters	Hemithyroidectomy, n=60	Total thyroidectomy, n=101	P-value
Age, years	55.6±13.5	56.2±14.6	NS
Range	26–90	26–80	
Females, %	83.4	88.1	NS
Follow-up, years	4.63±2.80	4.87±2.40	NS
Range	0.8–9.9	0.7–9.6	
Tumor size, mm	7.2±3.2	16.9±10.3	<0.001
Range	0.5–19.0	1.0–60.0	
Permanent complications, n (%)	3 (5.0)	5 (4.9)	NS
Visits to endocrine clinic per year, n	1.71±1.80	2.10±1.02	0.0160
Range	0.00–3.66	0.00–6.00	
Thyroid laboratory tests per year, n	3.04±2.0	3.21±1.5	NS
Range	0.1–12.0	0.0–9.8	
Neck ultrasounds per year, n	0.99±0.60	0.82±0.40	0.0320
Range	0.00–3.08	0.00–2.00	
FNAs during follow-up, n	1.00±1.31	0.11±0.35	<0.001
Range	0–5	0–2	
Neck reoperation, n (%)	4 (6.60)	1 (0.99)	0.065

Data are presented as mean ± standard deviation or number (%). FNAs, fine need aspirations; NS, not significant.
